# Analytical framework and data for a municipal solid waste environmental performance assessment

**DOI:** 10.1016/j.dib.2019.105085

**Published:** 2020-01-03

**Authors:** Rafael M. Deus, Fernando D. Mele, Barbara S. Bezerra, Rosane A.G. Battistelle

**Affiliations:** a São Paulo State University (UNESP), School of Engineering, Bauru, SP, Brazil; b National University of Tucumán (UNT), Department of Process Engineering and Industrial Management, San Miguel de Tucumán, Argentina

**Keywords:** Indicators, Strategic waste management, Environmental analysis, Carbon dioxide equivalent

## Abstract

This article contains (i) a set of spreadsheets with data compiled from municipal sanitation or solid waste plans, and (ii) data of the individual and aggregate performance indicators. These indicators have been published in the Journal of Cleaner Production in the article entitled “A municipal solid waste indicator for environmental impact: assessment and identification of best management practices.” The data contained in the spreadsheets are divided as follows: worksheet 1 includes the municipal solid waste generation data from the Brazilian municipalities studied; worksheet 2 presents the individual indicators that form the aggregate indicator; worksheet 3 presents the aggregate indicator and the classification of the municipalities; worksheet 4 provides data correlation; worksheets 5 to 10 depict boxplot graphs of the data; and worksheets 11 to 14 present graphs of individual indicators on a per capita basis and the ranking of municipalities.

**Table:** Specifications Table

Subject	Waste Management and Disposal		
Specific subject area	Municipal solid waste performance indicators		
Type of data	Spreadsheet		
How data were acquired	Data were acquired through content analysis of municipal solid waste or sanitary plans of 150 municipalities. After processing of raw data, the indicators for CO_2_ equivalent (CO_2_e) and energy consumption were acquired by modelling using the Waste Reduction Model.		
Data format	Raw and analyzed data, and concise numerical data.		
Parameters for data collection	A content analysis of 150 municipalities was conducted based on their waste management practices, as presented in municipal waste or sanitation plans, to calculate the greenhouse gases emissions and energy consumption of these municipalities. Based on the content analysis, the total amount of each type of waste destined for recycling, composting, and incineration (if applicable), and the total distance travelled by the waste to the landfill, composting, and recycling units were identified. These are secondary data that are available at governmental departments such as the Environmental Company of São Paulo State (CETESB), the Environment Department of São Paulo State, MSW or Basic Sanitation Plans, and the National System Information on Solid Waste Management.		
Description of data collection	The data were extracted based on a review of municipal solid waste or sanitary plans of municipalities. The data were then used to develop an aggregate indicator through a life cycle assessment approach.		
Data source location	The content of the solid waste and sanitary plans for the following small municipalities in São Paulo State, Brazil, were reviewed:		
	Águas da Prata Alambari Alfredo Marcondes Alvinlândia Anhembi Anhumas Aparecida D'oeste Arandu Arapeí Arco-Íris Arealva Areias Ariranha Aspásia Avaí Barão de Antonina Barra do Chapéu Barra do Turvo Bom Sucesso de Itararé Borá Boracéia Borebi Braúna Brejo Alegre Buritizal Cabrália Paulista Caiuá Campina do Monte Alegre Canas Canitar Catiguá Clementina Colômbia Coronel Macedo Corumbataí Cruzália Dirce Reis Dourado Dumont Echaporã Embaúba Espírito Santo do Turvo Estrela do Norte Estrela D'oeste Euclides da Cunha Paulista Flora Rica Gabriel Monteiro Gália Guatapará Herculândia Ibirarema	Icem Iepê Igarata Ilha Comprida Indiaporã Iporanga Irapuru Itaoca Itapirapuã Paulista Itapura Jambeiro Jeriquara João Ramalho Jumirim Lagoinha Lavrinhas Lucianópolis Luziânia Lutécia Macedônia Magda Manduri Mariápolis Meridiano Mesópolis Mira Estrela Monções Monte Alegre do Sul Monte Castelo Monteiro Lobato Motuca Nantes Narandiba Natividade da Serra Nova Aliança Nova Campina Nova Castilho Nova Guataporanga Nova Luzitania Ocauçu Óleo Onda Verde Oscar Bressane Palmeira D'oeste Paraiso Pardinho Parisi Paulistânia Paulo de Faria Pedrinhas Paulista Pereiras Platina Pongai	Pontalinda Pontes Gestal Populina Porangaba Presidente Alves Quadra Quintana Ribeira Ribeirão Corrente Ribeirão do Sul Ribeirão Grande Rifaina Sagres Salto Grande Santa Albertina Santa Clara D'oeste Santa Cruz da Conceição Santa Ernestina Santa Salete Santana da Ponte Pensa Santo Antônio da Alegria Santo Antônio do Araranguá Santo Antônio do Jardim Santo Antônio do Pinhal Santo Expedito Santópolis do Aguapeí São Joao do Pau D'alho São Jose da Bela Vista São Jose do Barreiro São Pedro do Turvo Sarapuí Sebastianópolis do Sul Silveiras Taciba Tapiraí Taquaral Taquarivaí Tarabai Tejupá Terra Roxa Torre de Pedra Torrinha Turiúba Ubirajara Uchoa Vitória Brasil
Data accessibility	The data are presented with this article.		
Related research article	The data are associated with the paper published online at the Elsevier journal “Journal of Cleaner Production”: Deus, R. M. et al. A municipal solid waste indicator for environmental impact: assessment and identification of best management practices [1].

**Table:** 

**Value of the Data** • The data can be used locally to assess the environmental impacts of municipal solid waste management in small municipalities in the state of São Paulo, Brazil. They may be used more broadly by those aiming at implementing efficient and effective aggregate indicators at other small municipalities. • The data benefit researchers who want to establish comparisons with their own data or use the data presented in this paper to develop and test new aggregate indicators. The data may also help public managers to elaborate better waste management policies aimed at improving the quality of public services provided to the population. • With minor adjustments or increments, such as the addition of new social and economic indicators, the data structure can be used to assess all dimensions of urban solid waste management sustainability, as well as to outline strategies for sustainable development.

## 1 Data

The datasheets contain data collected from municipal solid waste or sanitation plans. The datasheets also include data for the calculation of individual and aggregate indicators, which were collected from government and non-governmental organizations datasets. Timespan for the data is 1 year.

**Fig. 1:**
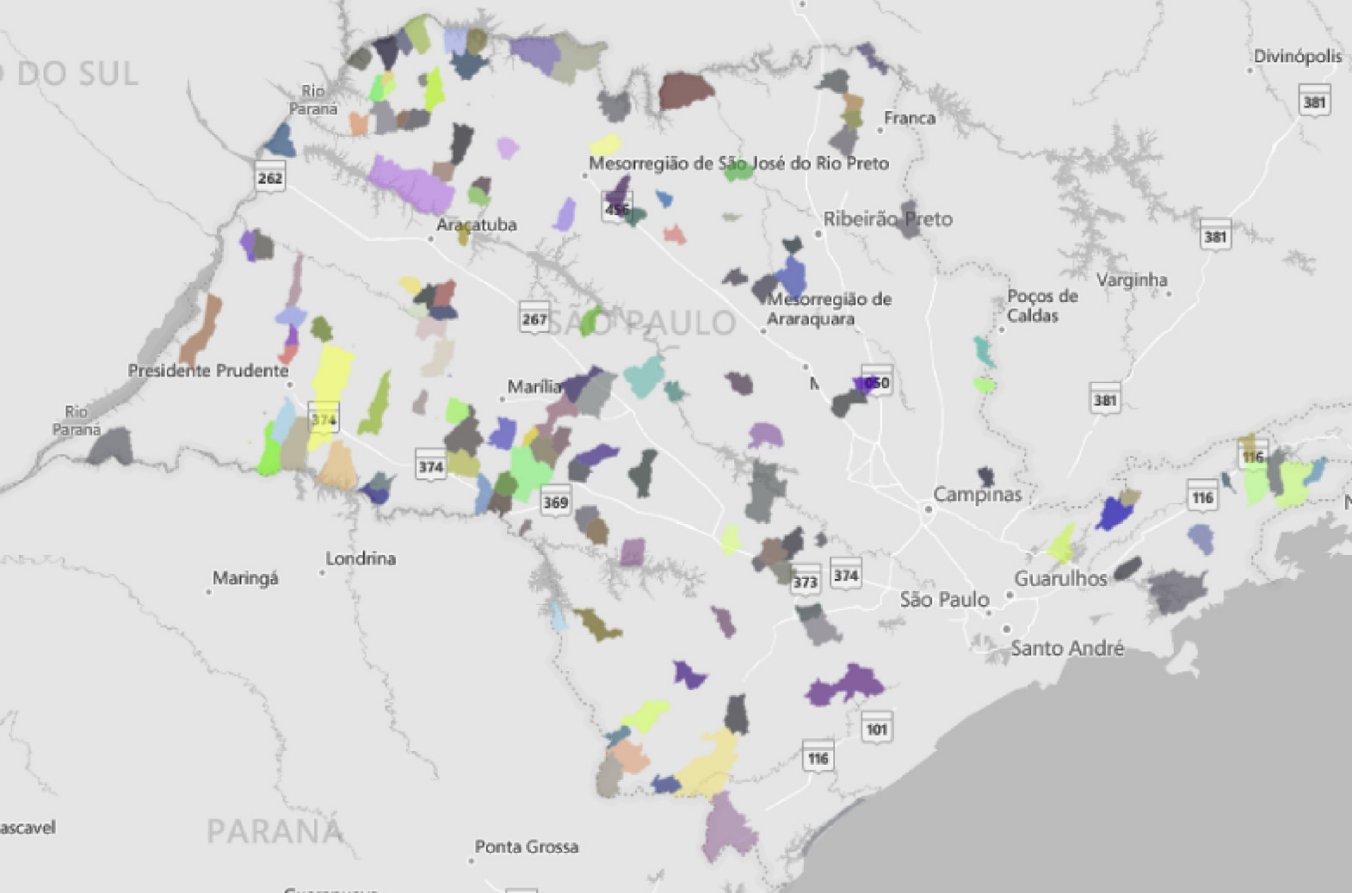
Small municipalities in the State of São Paulo included in this study.

Fig. 1 shows the municipalities that were considered in this study.

The article contains a spreadsheet data file (.xlsx format) with the following data tabs:

• Worksheet 1 (entitled “WASTE DATA”): Data about municipal solid waste generation and the average distance to the landfill and/or recycling/composting plant of 150 small municipalities (<10,000 inhabitants) in the state of São Paulo, Brazil;
• Worksheet 2 (entitled “INDICATORS”): Socioeconomic and individual indicators data resulting from the survey and modeling in the Waste Reduction Model (WARM);
• Worksheet 3 (entitled “MULTIPLE INDICATOR”): normalized individual indicators, aggregate environmental performance indicator and the classification of municipalities. This worksheet also contains two graphs, one referring to the general classification of municipalities, and the other referring to the composition of the normalized indicators of each municipality;
• Worksheet 4 (entitled “SPEARMAN CORRELATION”): Spearman correlation matrix between indicators. This worksheet also contains a graph of the correlations between variables;
• Worksheet 5 (entitled “ENERGY GRAPH”): descriptive summary of energy consumption (kWh) data and their outliers. This worksheet also contains a boxplot graph of the data;
• Worksheet 6 (entitled “CO2E GRAPH”): descriptive summary of carbon dioxide equivalent (CO_2_e) data and their outliers. This worksheet also contains a boxplot graph of the data;
• Worksheet 7 (entitled “POPULATION GRAPH”): descriptive summary of population data. This worksheet also contains a boxplot graph of the data;
• Worksheet 8 (entitled “GDP GRAPH”): descriptive summary of Gross Domestic Product (GDP) data and their outliers. This worksheet also contains a boxplot graph of the data;
• Worksheet 9 (entitled “WQI GRAPH”): descriptive summary of Waste Quality Index (WQI) data and their outliers. This worksheet also contains a boxplot graph of the data;
• Worksheet 10 (entitled “WASTE GENERATION GRAPH”): descriptive summary of waste generation data and their outliers. This worksheet also contains a boxplot graph of the data;
• Worksheet 11 (entitled “ENERGY RANK”): annual per capita indicator data and the ranking of municipalities for energy consumption. This worksheet also contains a graph of the ranking;
• Worksheet 12 (entitled “CO2E RANK”): annual per capita indicator data and the ranking of municipalities for CO_2_e. This worksheet also contains a graph of the ranking;
• Worksheet 13 (entitled “WQI RANK”): annual per capita indicator data and the ranking of municipalities for WQI. This worksheet also contains a graph of the ranking;
• Worksheet 14 (entitled “WASTE GENERATION RANK”): annual per capita indicator data and the ranking of municipalities for waste generation. This worksheet also contains a graph of the ranking;

## 2 Experimental design, materials, and methods

### 2.1 Selection of municipalities

This study adopted a secondary data extraction method to obtain data from small municipalities (up to 10,000 inhabitants) in the State of São Paulo. The inclusion criteria consisted of all necessary data for the performance of life cycle assessment, such as waste generation, composition, recycling and composting rate, being available for the municipality. Of a total of 645 municipalities in São Paulo State, 273 are small municipalities, and of these, a total of 123 municipalities were excluded from the sample due to unviable, corrupted, vitiated, unrealistic, or partially available data, resulting in 150 municipalities being analyzed (Fig. 1).

### 2.2 Data collection

Each municipality considered was analyzed based on the waste management practices included in its municipal plans of basic sanitation or solid waste plans. Data was gathered in structured spreadsheets and they present the total amount of each type of waste and their destination, the amount of waste that is recycled and composted, and the distance traveled by the waste to the landfill, composting, and/or recycling units. The general gravimetric composition of municipal solid waste was collected from solid waste plans for small municipalities in São Paulo State.

### 2.3 Waste Reduction Model

For assessment of individual indicators data, the Waste Reduction Model (WARM), Version 14, from the United States Environmental Protection Agency, a widely applied model in many different cities around the world [[2], [3], [4], [5], [6]], was used to process the data.

### 2.4 Indicators data (outputs)

At the final stage, the environmental impacts data of municipalities are presented in the spreadsheets as CO_2_ equivalent, which comprises the set of gases that contribute to global warming, and the energy consumption [7].

### 2.5 Aggregate indicator

An aggregate data performance indicator is presented in the spreadsheet. The components of the aggregate indicator are the per capita waste generation, which is widely used [6], the per capita emission of CO_2_e, the per capita energy consumption, and the Waste Quality Index (WQI), developed by CETESB [8]. The WQI evaluates final disposal sites for solid waste from a technical and environmental point of view. Its value may range from 0.0 to 10.0 points, with values below 7.0 points indicating poor landfill conditions, while points ranging from 7.1 to 10.0 indicate sufficient landfill conditions.

### 2.6 Calculating the aggregate indicator

Each indicator (I) was normalized using the relative maximum and minimum values (Equation (1)) from the data collected and presented in the spreadsheet. The normalization generated indicators with values ranging from 0 to 1 [1].

(1) $$I=\frac{x_{i}-x_{i,min}}{x_{i,max}-x_{i,min}}$$

where x_i,max_ and x_i,min_ are the maximum and minimum values based on the data, for each indicator (i), respectively.

After normalization, the aggregation was performed by computing the geometric mean of the normalized indicators, according to Equation (2). All indicators were given equal weight (1/4). The aggregate indicator (AI) varies between 0 and 1.

(2) $$AI=\;\sqrt[{4}]{WG_{I}\times GHG_{I}\times EC_{I}\times WQI_{I}}$$

where WG_I_ is the normalized indicator of solid waste generation (kg inhabitant^−1^ year^−1^), GHG_I_ is the normalized indicator of CO_2_e emission, EC_I_ is the normalized indicator of energy consumption and WQI_I_ is the normalized indicator of Waste Quality Index (WQI).

All collected and processed data are presented in the spreadsheet.
